# Communication and visiting policies in Italian intensive care units during the first COVID-19 pandemic wave and lockdown: a nationwide survey

**DOI:** 10.1186/s12871-022-01726-1

**Published:** 2022-06-17

**Authors:** Thomas Langer, Francesca Carmela Depalo, Clarissa Forlini, Silvia Landini, Andrea Mezzetti, Paola Previtali, Gianpaola Monti, Carolina de Toma, Davide Biscardi, Alberto Giannini, Roberto Fumagalli, Giovanni Mistraletti, on behalf of the COMVISCOV group, Barbara Lissoni, Andrea De Martini, Nadia Mareto, Concetta Rossitto, Ugo Zummo, Martina Taverna, Patrizia Machieraldo, Mauro Navarra, Massimiliano Parlanti Garbero, Chiara Scaletti, Silvia Perno, Luca Amendolia, Giorgia Montrucchio, Deliana Veliaj, Giuseppe Barbarello, Maria Alesci, Luca Bolgiaghi, Davide Vailati, Angelo Pezzi, Enrico Boselli, Francesca Piccoli, Massimiliano Greco, Marco Gemma, Marco Resta, Stefania Crotti, Nicola Bottino, Chiara Abruzzese, Monica Savioli, Giuseppina Migliorino, Stefano Muttini, Michele Umbrello, Beatrice Borghi, Stefano Greco, Micaela Dizeo, Maurizio Bottiroli, Michele Giovanni Mondino, Manlio Prosepri, Giampaolo Casella, Francesco Curto, Matteo Zaniboni, Riccardo Giudici, Carlo Gentile, Michela Bombino, Roberto Rona, Barbara Cortinovis, Annalisa Benini, Leonello Avalli, Mario Tavola, Matteo Ferrario, Roberta Preda, Enzo Primerano, Gianluca Russo, Virginia Porta, Federico Valdambrini, Paola Fassini, Serena Orando, Eduardo Beck, Matteo Pedeferri, Giacomina Cogliati, Denise Testini, Benedetta Moroni, Vito Codeluppi, Patrizia Ruggeri, Elisa Milanesi, Mirko Belliato, Alessandra Besozzi, Mario Riccio, Silvia Zerbi, Davide Corbella, Francesco Ferri, Lorenzo Grazioli, Ezio Bonanomi, Matteo Giacomini, Noemi Sacchi, Cristian Codognola, Alessandra Ambrosini, Luca Guatteri, Matteo Subert, Gian Paolo Castelli, Massimo Borelli, Erica Venier, Loredana Dittura, Stefania Buttera, Roberto Bigai, Sandra Magnoni, Simon Rauch, Angelo Colombo, Giorgio Fullin, Caterina Donolato, Silvia Cattin, Veronica State, Enrico Redeghieri, Alessandro Russo, Simonetta Pastorini, Sandra Allena, Marina Munari, Federica Turchet, Mario Peta, Vincenzo De Santis, Cristina Scala, Francesca Facondini, Elisabetta Marangoni, Tania Tassinati, Chiara Zanzani, Emanuele Russo, Annamaria Marchio, Maria Barbagallo, Massimo Girardis, Paolo Taffache, Marco Mordacci, Matteo Vincenzi, Michele Pennica, Giovanna Bracciotti, Paola Iori, Davide Gambi, Iacopo Cappellini, Lara Vegnuti, Alessandra De Luca, Stefano Romagnoli, Giamila Mosti, Rossella Carla, Valeria Roticiani, Lorella Pelagalli, Ennio Fuselli, Emilio D’Avino, Massimo De Bellis, Giulia Gianni, Francesca Leonardis, Marzia Rossi, Rossana Lorusso, Eugenia Magnanimi, Sabrina Martelli, Floriana Baisi, Davide Balsamo, Virginia Cotticelli, Alessia Mattei, Ivano Farinelli, Teresa Riccini, Luisanna Cola, Antonella Jorio, Emanuele Iacobone, Roberta Domizi, Simone Pizzi, Armando Nasso, Romano Graziani, Anna Monaco, Manuela Manno, Carla Maria Ottelio, Michela Del Rio, Antonio Serra, Barbara Enna, Francesco Marco Loddo, Rita Galbiati, Serena Mellea, Michelle Brozzi Kimberly, Matteo Vissani, Francesco Massimo Romito, Laura Baccari, Nadia Zarrillo, Clelia Esposito, Patrizia Murino, Salvatore Notaro, Carmine Ausiello, Annachiara Marra, Carmela Policastro, Chiara Cafora, Giuseppe De Benedectis, Vincenzo Di Falco, Maria Sciddurlo, Giancarlo Negro, Paolo Vetuschi, Andrea Recchia, Rita Pasquariello, Rosalba Squillace, Antonio Ciambrone, Carmela Bencivenga, Melania Camiolo, Cristina Agozzino, Francesco Oliveri, Tiziana Notarrigo, Giacomo Castiglione, Antonella Mo, Laura Condorelli, Martina Favarato

**Affiliations:** 1 Department of Medicine and Surgery, University of Milan-Bicocca, Monza, Italy; 2 Department of Anesthesia and Intensive Care Medicine, Niguarda Ca’ Granda, Milan, Italy; 3 Azienda USL Toscana Centro, 118 Empoli, Empoli, Italy; 4 Department of Anesthesia and Intensive Care, ASST Santi Paolo e Carlo, San Paolo University Hospital, Milan, Italy; 5 Unit of Pediatric Anesthesia and Intensive Care, Children’s Hospital, ASST Spedali Civili, Brescia, Italy; 6 Department of Pathophysiology and Transplantation, University of Milan, Milan, Italy

**Keywords:** Pandemics, Intensive care units, Health communication, Professional-family relations, Patient-centered care

## Abstract

**Background:**

During the first coronavirus disease 2019 (COVID-19) pandemic wave, an unprecedented number of patients with respiratory failure due to a new, highly contagious virus needed hospitalization and intensive care unit (ICU) admission. The aim of the present study was to describe the communication and visiting policies of Italian intensive care units (ICUs) during the first COVID-19 pandemic wave and national lockdown and compare these data with prepandemic conditions.

**Methods:**

A national web-based survey was conducted among 290 Italian hospitals. Each ICU (active between February 24 and May 31, 2020) was encouraged to complete an individual questionnaire inquiring the hospital/ICU structure/organization, communication/visiting habits and the role of clinical psychology prior to, and during the first COVID-19 pandemic wave.

**Results:**

Two hundred and nine ICUs from 154 hospitals (53% of the contacted hospitals) completed the survey (202 adult and 7 pediatric ICUs). Among adult ICUs, 60% were dedicated to COVID-19 patients, 21% were dedicated to patients without COVID-19 and 19% were dedicated to both categories (Mixed). A total of 11,102 adult patients were admitted to the participating ICUs during the study period and only approximately 6% of patients received at least one visit. Communication with family members was guaranteed daily through an increased use of electronic devices and was preferentially addressed to the same family member. Compared to the prepandemic period, clinical psychologists supported physicians more often regarding communication with family members. Fewer patients received at least one visit from family members in COVID and mixed-ICUs than in non-COVID ICUs, l (0 [0–6]%, 0 [0–4]% and 11 [2–25]%, respectively, *p* < 0.001). Habits of pediatric ICUs were less affected by the pandemic.

**Conclusions:**

Visiting policies of Italian ICUs dedicated to adult patients were markedly altered during the first COVID-19 wave. Remote communication was widely adopted as a surrogate for family meetings. New strategies to favor a family-centered approach during the current and future pandemics are warranted.

**Supplementary Information:**

The online version contains supplementary material available at 10.1186/s12871-022-01726-1.

## Background

Italy was the first European country hit by the coronavirus disease 2019 (COVID-19) pandemic [1–3]. COVID-19 has a wide range of clinical presentations, including the acute respiratory distress syndrome (ARDS) [4, 5]. An extremely high number of subjects developed ARDS resulting in a sharp increase in intensive care unit (ICU) admissions [6]. This rapid and unforeseen surge of critically ill patients required a quick increase in ICU beds, highly stressing the health care system [7].

At that time, very little was known about COVID-19, therapy consisted mainly of supportive care [8] and the mortality of critically ill patients was very high [2, 9]. Moreover, the highly contagious nature of the virus was evident, and most countries enforced very strict lockdowns/“Stay-at-Home orders” to reduce viral spread. These measures included restriction of hospital visits both for COVID-19 patients and for patients hospitalized for other reasons. The aim was to reduce the possibility of family members being infected in the hospital, and to reduce the possibility of family members being vehicles of infection.

Normally, that is, outside of a pandemic caused by a new and highly contagious virus, there is no scientific basis for limiting family presence in the ICU [10]. Indeed, an “open” ICU policy, in addition to recognizing a specific and unequivocal right of the patient, is also a useful and effective strategy to respond to the needs of both patients and families [10, 11]. Moreover, an unrestricted visiting policy produces consistent positive effects on family member anxiety and depression symptoms [12], supports family-centered care, including shared decision-making [13], improves the relations and communication of ICU staff with families, and favors trust and appreciation from family members [14]. During the first COVID-19 pandemic wave, the lack/reduction of ICU visits forcedly changed the communication habits, shifting from well-established family meetings [15, 16], to remote communications using electronic devices [17, 18].

Both health care professionals and the general public witnessed these dramatic changes [19–22], and the literature on this topic is growing [23–29]. While several studies focused their attention on ICUs dedicated to patients with COVID-19, less information is available regarding the impact on ICUs dedicated to patients without COVID. Moreover, no reports thus far have assessed the role of clinical psychology during the pandemic wave. Overall, clinical psychology is increasingly recognized as a fundamental part of critical care [30], aimed at supporting critical care patients, their families and the ICU staff, both singularly and during the interactions occurring in family meetings.

The aims of the present study were therefore to i) describe how Italian hospitals changed their organization during the first pandemic wave; ii) describe the changes in ICU communication and visiting policies and iii) investigate the role of clinical psychology in this context.

## Methods

A national web-based survey was conducted between December 10, 2020 and February 2, 2021 among hospitals participating in the “Intensiva 2.0” project [31], approved by the Ethical Committee (Comitato Etico Milano Area A, protocol number 35410_2017). This project was born from www.intensiva.it, an Italian project promoting a human-centered intensive care [32]. After consultation with the Ethical Committee, the need for an additional ethics approval was waived, given the nature of the study and the previous approval of the project. Participants responded to the survey on a voluntary basis. The response to the survey was considered a written consent to participate.

The survey was developed by the authors and a pilot test [33] was performed in 5 hospitals in Lombardy. Thereafter, 290 hospitals with at least one ICU prior to December 2020 were contacted. The survey was announced with a newsletter. Subsequently physicians/nurses personally participating in the “Intensiva 2.0” project were contacted via email and/or phone calls (up to three times) to increase the response rate. Indications were given to complete the questionnaire with the ICU coordinator. Each ICU that was active between February 24 and May 31, 2020 was encouraged to complete an individual questionnaire.

The following information regarding the hospitals was retrieved from the Intensiva 2.0 database: university affiliation, trauma center, and presence of a room for family conferences. The number of hospital beds was retrieved from official governmental sources (http://www.dati.salute.gov.it). Hospital size was categorized according to the number of beds (< 250; 250–424; > 425 beds) [34]. The survey did not contain information about patients, or sensitive personal data.

### Survey questionnaire

The survey consisted of single- and multiple-choice questions structured into 5 sections. The English translation of the survey is reported in the Online Supplementary Material (OSM). The *first section* investigated the organization/structure of the ICU and hospital prior to (prepandemic) and during the first pandemic wave (pandemic), including information regarding the type of ICU, i.e., dedicated to patients with COVID-19 (COVID-ICU), without COVID-19 (non-COVID-ICU) or to both categories (mixed-ICU). This section included a question (number 13) regarding the number of treated patients during the study period. Answers were categorical (e.g. 21–30 patients, 31–40 patients). If more than 100 patients were treated, the precise number was needed. To obtain a quantitative estimate, we used the average for each category. For example, for ICUs selecting 31–40 patients, we considered 36 treated patients. The responding ICUs were divided into pediatric ICUs and adult ICUs and analyzed separately.

The *second* and *third sections* aimed to describe the habits regarding family-patient and family-physician communications. The *fourth section* focused on the role of the clinical psychology service, while the *last section* investigated the ICU visiting policies. This section included a question (number 8) regarding the number of patients who received at least one visit during their ICU stay during the study period. Answers were categorical (e.g.*,* 11–15 patients, 16–20 patients). If more than 30 patients received at least 1 visit, the precise number was needed. To have a reasonable estimate of the number of patients who received at least one visit, we used the average for each category. For example, for ICUs selecting the category “16–20 patients” we considered that 18 patients had received at least one visit.

### Statistical analysis

Continuous variables were summarized as medians and interquartile ranges, and categorical data were summarized as counts and percentages. Mann-Whitney rank-sum test and χ2 or Fisher’s exact test were used to compare nonparametric continuous variables and categorical variables, respectively. One-way ANOVA on ranks was used to compare continuous data from COVID, non-COVID and Mixed ICUs. All statistical tests were 2-tailed, and statistical significance was defined as *p* < 0.05. Analyses were performed using the statistical package STATA 16.0 (StataCorp LLC, College Station, TX, USA) and SigmaPlot 11.0 (Systat Software Inc., San Jose, CA, USA).

## Results

Two hundred and nine questionnaires from 154 hospitals (53% of the 290 contacted hospitals) were completed for the present survey and were included in the study. Table 1 summarizes the prepandemic characteristics of the responding hospitals. Differences from the 136 nonesponding hospitals are summarized in Table E1 of the OSM. The geographic distribution of the responding ICUs is reported in Fig. E1. Of note, a geographic imbalance regarding responding hospitals was observed, with more centers from northern Italy answering the questionnaire. Of the responding ICUs, 202 (97%) were adult ICUs, while 7 (3%) were pediatric ICUs.

**Table 1:** Characteristics of participating Hospitals

Variables	Hospitals (*n =* 154)
University affiliated hospital - no. (%)	36 (23)
Trauma center - no. (%)	49 (32)
Hospital beds - no. (%)	
**<** 250	43 (28)
250–424	49 (32)
**>** 425	62 (40)
Hospital ICU beds - no. (%)	
< 20 beds	115 (75)
> 20 beds	39 (25)
Dedicated room for family meetings - no. (%)	125 (81)
Visiting-hour policies* - no. (%)	
≤ 2 hours per day	64 (38)
3–6 hours per day	41 (24)
7–12 hours per day	45 (26)
> 12 hours per day	20 (12)

Table 1 Table summarizes the preandemic characteristics of the 154 participating hospitals. * refers to 170 intensive care units, i.e. each participating hospital could have more intensive care units with potentially different visiting policies.

### Hospital reorganization

During the first pandemic wave, the number of ICUs and the number of ICU beds per hospital increased significantly (from 1 [1 - 2] to 2 [1 - 3] ICUs, *p* < .001 and from 9 [6 - 20] to 20 [10 - 33] ICU beds, p < .001, respectively). To face this surge, 109 (71%) hospitals increased the number of medical staff working in the ICU: 32 (29%) hospitals hired new board-certified intensivists; 63 (58%) hospitals hired senior intensive care residents; 109 (93%) relocated staff anesthesiologists from the operating room; and 9 (8%) relocated specialists from other disciplines to the ICU. Several hospitals applied a combination of these strategies. As a result, the average daily intensivist-to-bed ratio did not change significantly during the first pandemic wave (5.8 [4.4–6.7] to 5.4 [4.2–7.2], *p* = 0.572).

Similarly, 124 (81%) of the responding hospitals increased the number of ICU nurses. This was achieved through the hiring of new personnel in 65 (26%), relocation of nurses from the operating room in 109 (88%) and relocation of nurses from regular surgical/medical wards in 75 (60%) hospitals. Again, a combination of these strategies was frequently adopted.

### Intensive care units dedicated to adults

Out of the ICUs dedicated to adults, 121 (60%) were COVID-ICUs, 43 (21%) were non-COVID-ICUs and 38 (19%) were mixed-ICUs.

Overall, we reported information regarding 2524 ICU beds for adult patients: 1546 for COVID-19 patients, 348 for patients without COVID-19 and the remaining 630 beds for both categories.

During the study period, the included hospitals admitted approximately 11,102 patients: 5857 (53%) patients were admitted to COVID-ICUs, 2822 (25%) patients were admitted to non-COVID ICUs, and 2423 (22%) patients were admitted to to mixed-ICUs.

The first pandemic wave had a major effect on visiting policies (Table 2). Prior to the pandemic, daily visits were allowed in all participating ICUs, while this was possible only in 4% of ICUs during the first pandemic wave. Moreover, in 103 (51%) of the responding ICUs family members were never allowed in the ICU (Table 2). In addition, while physical contact during visits was allowed in 98% of the ICUs prior to the pandemic surge, this was allowed only in 28% during the pandemic (*p* = .0001). Changes were also reported regarding the family members who could visit, with access preferentially given to the same family member during the pandemic, while more liberal access was described prior to the pandemic. Overall, approximately 673 patients received at least one visit during the first pandemic wave. Considering the number of treated patients, this corresponds to 6% of visits. The reasons reported by the participating ICUs for the lack/marked reduction of visits to the ICU were several (multiple choice) and included hospital visit ban (187, 89%), national lockdown (32, 15%), family self-quarantine (22, 11%), refusal by family members (5, 2%) and ICU habits (8, 4%).

**Table 2:** Changes in visiting policies in adult ICUs

	Prepandemic	First pandemic wave	***P-***value
**Permission to visit patients** - no. (%)			
Daily	163 (100)	8 (4)	< 0.001
2–3 times per week	0	10 (5)	
For major events only	0	81 (40)	
No	0	103 (51)	
**Physical contact allowed**	159 (98)	28 (28)	< 0.001
**Which family member visits?** - no. (%)	/161	/57	< 0.001
Mainly the same family member	13 (8)	36 (63)	
Mainly the same family members	99 (61)	15 (26)	
Any family member	30 (19)	4 (7)	
Anyone	19 (12)	2 (4)	

Table 2 Table summarizes the ICU visiting policies of the participating centers. The prepandemic period refers to 163 ICUs, while the first pandemic wave refers to 202 ICUs. The percentage of the third item refers to the responding ICUs that allowed some visits in the ICU.

Table 3 summarizes the major findings regarding the changes regarding communication habits caused by the first pandemic surge. The use of electronic devices for remote communication increased significantly (95% vs. 75%, *p* = .0001) to favor remote *family-physician* communication. In particular, for this purpose, there was an increased use of video calls. As a result, the timing of communication did not change significantly, as daily information regarding the patients’ clinical conditions was provided to the family in both study periods. Similarly, we did not observe significant variations regarding the person providing clinical information and her or his communication expertise. In contrast, there was a significant change regarding the family member receiving the information: during the first pandemic wave, information was preferentially provided to the same family member/members.

**Table 3:** Changes in communication habits in adult ICUs regarding physician-family and patient-family communication

	Prepandemic	First pandemic wave	***p-***value
**Use of electronic devices for physician - family communication - n (%)**	123 (75)	191 (95)	< 0.001
**Kind of electronic device used**	/123	/191	< 0.001
Voice call - no. (%)	108 (88)	152 (75)	
Video call - no. (%)	21 (17)	105 (52)	
Other device - no. (%)	10 (8)	11 (5)	
**Communication with families occurs**			> 0.999
Daily - no. (%)	162 (99.4)	199 (98.5)	
2–3 times per week - no. (%)	1 (0.6)	2 (1)	
For major events only - no. (%)	0	1 (0.5)	
**Who gives information to the family members?**			0.197
Mainly the same person - no. (%)	31 (19)	43 (21)	
Mainly the same persons - no. (%)	63 (39)	60 (30)	
Whoever was in charge of the patient - no. (%)	69 (42)	99 (49)	
**Physician’s giving information are**			0.166
experienced in communication - no. (%)	115 (71)	128 (63)	
both experienced and not experienced - no. (%)	48 (29)	74 (37)	
**The doctor gives information:**			< 0.001
mainly to the same family member - no. (%)	53 (33)	116 (57)	
mainly to the same family members - no. (%)	82 (50)	75 (37)	
to any family member - no. (%)	26 (16)	8 (4)	
to anyone - no. (%)	2 (1)	3 (2)	
**Use of electronic devices for patient- family communication - n (%)**	83 (51)	162 (80)	< 0.001
**Patients have free access to personal electronic devices - n (%)**	101 (62)	188 (93)	< 0.001
**Communication between patient and family occurs**			< 0.001
Daily - no. (%)	147 (90)	132 (65)	
2–3 times per week - no. (%)	7 (4.5)	33 (16)	
On occasion - no. (%)	7 (4.5)	25 (12)	
Never - no. (%)	2 (1)	12 (6)	

Regarding *patient-family* communication, despite an increased use of electronic devices for this purpose (51% vs. 80%, *p* < 0.001) and an increase in patients’ free access to personal electronic devices (62% vs. 93%, *p* < 0.001), communication between patients and relatives was significantly reduced during the first pandemic wave.

The percentage of ICUs supported by a clinical psychology service did not change significantly (Table E2); however, during the first pandemic wave psychologists assisted physicians more often with family communication (20 vs. 30%, *p* = 0.031). Moreover, the modalities through which the clinical psychologists interacted with family members changed, with an increase in indirect assistance through phone calls (Table E2). The same was true regarding the assistance to patients: during the pandemic wave, the interaction between psychologists and patients was mainly indirect through the attending physician.

### Comparison between COVID, non-COVID and mixed-ICUs

**Fig. 1:**
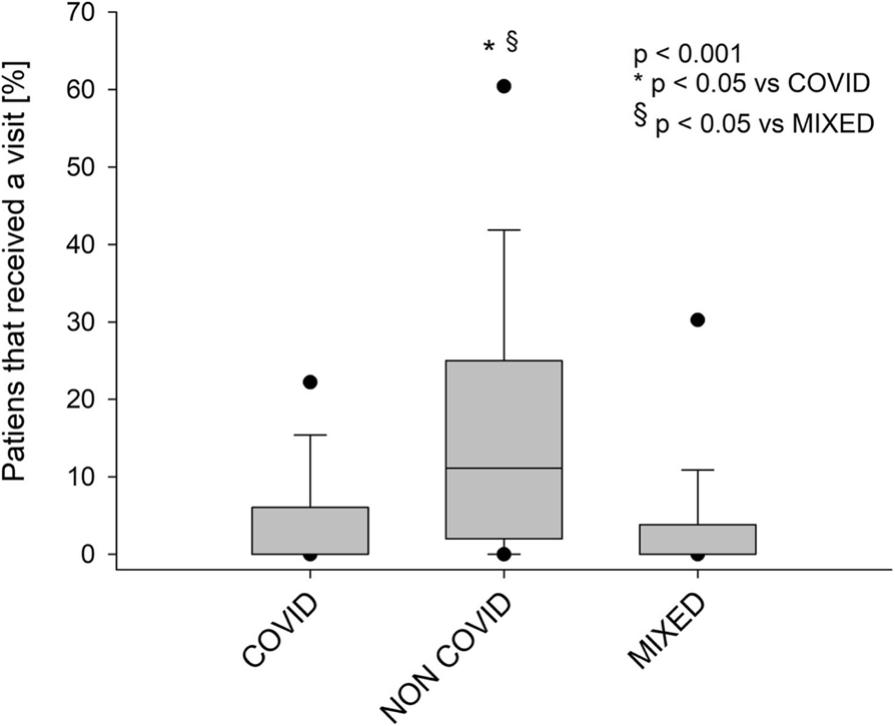
Percentage of patients receiving at least one visit according to the type of intensive care unit. A one-way ANOVA was conducted (*p <* 0.001)

When comparing different types of ICUs during the first pandemic wave, a significant difference regarding the percentage of patients receiving at least one visit was observed (Fig. 1). In particular, the median value of patients receiving a visit was close to zero and similar for COVID (0 [0–6]%) and mixed- (0 [0–4]%) ICUs, while it was 11 (2–25%) for non-COVID ICUs. Moreover, we observed that more COVID- and mixed-ICUs used electronic devices for patient-family communication and allowed free access to personal electronic devices. Details regarding the differences between these three categories of ICUs are reported in Table 4.

**Table 4:** Comparison between COVID, Non-COVID and Mixed ICUs

Variable	COVID (***n =*** 121)	Non-COVID (***n =*** 43)	Mixed (***n =*** 38)	***p-***value
**Use of electronic devices for physician - family communication - n (%)**	115 (95)	40 (93)	36 (95)	0.908
**Kind of electronic device used**	/115	/40	/36	0.680
Voice call	101 (88)	34 (85)	25 (70)	
Video call	67 (58)	17 (42)	21 (58)	
Other devices	6 (5)	3 (8)	2 (6)	
**Communication with families occurs**				0.267
Daily - no. (%)	120 (99)	42 (98)	37 (97)	
2–3 times per week - no. (%)	1 (1)	0	1 (3)	
For major events only - no. (%)	0	1 (2)	0	
**The doctor gives information**				0.046
mainly to the same family member - no. (%)	79 (65)	18 (42)	19 (50)	
mainly to the same family members - no. (%)	37 (31)	20 (47)	18 (47)	
to any family member - no. (%)	4 (3)	3 (7)	1 (3)	
to anyone - no. (%)	1 (1)	2 (5)	0	
**Use of electronic devices for patient-family communication – n (%)**	107 (88)	24 (56)	31 (82)	< 0.001
**Patient has free access to personal electronic devices**	115 (95)	36 (84)	37 (97)	0.037
**Communication between patient and family occurs**				0.763
Daily - no. (%)	79 (65)	26 (60)	27 (71)	
2–3 times per week - no. (%)	21 (18)	7 (16)	5 (13)	
On occasion - no. (%)	16 (13)	6 (14)	3 (8)	
Never - no. (%)	5 (4)	4 (10)	3 (8)	

### Pediatric intensive care units

From the 7 pediatric ICUs, 3 were exclusively dedicated to children without COVID-19, 4 were mixed ICUs, and none were exclusively dedicated COVID-19. We reported data on 22 ICU beds dedicated to non-COVID patients and 46 mixed ICU beds. Overall, an approximate number of 263 children (159 non-COVID and 104 to mixed-ICUs) were admitted to the participating pediatric ICUs during the study period.

Visiting was always guaranteed to at least one parent/caregiver during the first pandemic wave. No significant variations regarding visiting and communication habits were observed (Table E3).

## Discussion

We conducted a national, multicenter survey in Italy, which included information from 209 ICUs of 154 Italian hospitals. Our aim was to analyze the changes caused by the first COVID pandemic wave regarding hospital/ICU organization and ICU visiting/communication habits. The main findings are that hospitals rapidly reorganized to face the surge in critically ill patients with respiratory failure due to a new, highly contagious viral disease. Major limitations were put in place regarding ICU visiting policies, with the aim of reducing interpersonal contact and the consequent risk of viral spread. These interventions affected the ICU visiting policies and family-physician communication in a dramatic and unprecedented way. Indeed, the familial presence in the ICU, a fundamental part of the humanization process with clinical implications of the utmost importance [35–37], ceased almost completely. Moreover, well-established family meetings [38–40], a crucial clinical moment to exchange information and build a trustworthy and collaborative relationship, could not take place. Nevertheless, physicians adapted to the new and challenging situation, providing daily information to family members through remote communication. Finally, clinical psychologists more often supported physicians in this challenging task.

Hospitals that participated in the present study were from all over Italy, although approximately 30% were concentrated in Lombardy, a region in the northern part of the country. This finding has several explanations. First, Lombardy has more than 10 million inhabitants, approximately one-sixth of Italy’s population. Second, it has a very broad health care system, and finally, during the first pandemic wave it was the most affected region (Fig. E1). Overall, Italian hospitals significantly increased their ICU capacity and staffing. In this way, an unprecedented number of critically ill patients could be treated simultaneously [7] without significantly reducing the intensivist-to-bed ratio. We estimated that the participating ICUs cared for more than 11,000 patients during the first pandemic wave.

Increasing attention is given to the presence of family members in the ICU [41, 42]. In the past, there have been many objections to liberalizing visiting policies in ICUs. Indeed, for a long time, the family was considered a burden, a possible source of infection and disturbance for the staff. The presence of family members was considered stressful for the patient and for the relatives. There was, however, no scientific basis for limiting the presence of family in the ICU [14]. An Italian survey conducted by Giannini et al. in 2008 described that the median daily ICU visiting time was only approximately 1 hour [42]. The same authors described in 2011 some improvement, with a median visiting time of approximately 2 hours [43]. Our prepandemic starting point underlines that, prior to the pandemic, further progress was achieved in Italian ICUs. Indeed, daily visits to patients were allowed in all ICUs, with a median visiting time of 5 [2 -10] hours and with 12% of ICUs allowing family access more than 12 hours per day (Table 1).

The outbreak of the new betacoronavirus and the consequent national lockdown changed these habits immediately, putting the advances regarding visit liberalization gained with difficulties in recent years at risk. Indeed, all hospitals strictly limited family access to hospitals and ICUs, and almost 90% of hospitals prohibited access to family members. While not directly investigated in our survey, we think that clinicians, directly facing the human tragedies, sometimes disobeyed the restrictions and permitted access to the ICU [26]. We estimated that only 6% of the more than 11,000 patients treated in the ICU during the study period received at least one visit. Given the high mortality of COVID-19 and, in general, of critical illness, this means that thousands of relatives could not be close to their loved ones, even during the most delicate moments, such as the end of life [44–46]. Moreover, in addition to the denied access to the ICU, the few relatives that could visit were frequently asked to avoid physical contact with their loved ones (Table 2).

The percentage of patients receiving a visit was different according to the type of ICU, i.e.*,* patients admitted to “non-COVID ICUs” received more visits than patients admitted to COVID or mixed-ICUs (Fig. 1). This finding clearly underlines that the risk of viral transmission was bilateral, i.e., family members could inadvertently be asymptomatic carriers and infect their loved ones in non-COVID ICUs, but physicians and hospitals were also concerned that family members could contract the virus in the ICU. In addition, a complex gowning procedure and the use of dedicated personal protective equipment (PPE) were required to access COVID-ICUs. It is important to emphasize that the family needed to be assisted/supervised by health care workers during the gowning and subsequent removal of PPE. Gowning procedures were frequently required in the past to access the ICU [47], with the purpose of limiting infection transmission from the relative to the patient. This habit was partially abandoned in recent years due to a lack of evidence. However, during the first pandemic wave, PPE became fundamental. There was a worldwide scarcity of high-filtration masks and other PPE, forcing, on the one hand, to reuse masks designed for single use [48], and, on the other hand, further complicating the ICU access to family members. From an ethical point of view, the shortage of PPE is one of the few acceptable reasons to limit family presence, as PPE for health care professionals is a priority to guarantee the treatment of patients [49].

Receiving clear, understandable and timely clinical information is fundamental for family members [13, 38, 50, 51]. The physical absence of relatives from the ICU forced clinicians to completely change their communication habits. Indeed, as family meetings could not be conducted in person, electronic devices were widely adopted as a surrogate. The broad and increased use of phone and video calls guaranteed daily communications (Table 3). The use of phone calls to communicate with the family of ICU patients is not new [42, 47]. However, the purpose in the prepandemic period was likely different. Indeed, in the past, phone calls were mainly as a complementary tool to family meetings to provide reassurance to family members. In contrast, during the pandemic wave, they were a necessary alternative, a surrogate for family meetings.

Interestingly, we observed that information was preferentially provided to the same family member during the pandemic, as opposed to the prepandemic period. This was very likely an attempt to establish a trustworthy relationship despite obvious difficulties and to avoid possible misunderstandings and fragmented communication due to multiple interlocutors. It is important to emphasize that, despite the evident effort to provide remote communication in an extremely difficult context, the effectiveness of such communication modalities remains a matter of debate [52]. Furthermore, the communication challenges could enhance ethical conflicts in the ICU and, consequently health care providers’ distress [53]. In particular, “surrogate” remote-communication risks increasing the challenges of shared decision-making and the quality of care in end-of-life [44, 54]. The impact of the modified visiting and communication policies on these aspects deserves further attention in future studies.

While the presence of clinical psychologists in the ICU did not increase significantly, we observed that their role somehow changed. Indeed, given the physical absence of family members, clinical psychologists more frequently assisted clinicians with the challenging task of remote communication. In light of this experience and the growing literature on the subject, there is, in our opinion, the need to increase the presence of clinical psychologists in the ICU.

Finally, it is important to note, that the pediatric ICUs participating in the study reported only minor disruptions regarding visiting and communication habits. In this particular context, i.e., the care of critically ill children, the presence of the parents is (and is increasingly perceived) as fundamental [55, 56]. Of course, the experience of pediatric ICUs strongly suggests that opening the ICU during a pandemic of respiratory disease is feasible.

### Limitations

Our study has several limitations. First, participating ICUs adhered to a project designed to improve communication and humanization in the ICU [31]. It is therefore conceivable that our population is somehow biased and that the overall Italian reality might be somehow different. Moreover, we had a response rate of 53% with a relevant geographic imbalance, which could be a further cause of selection and cultural bias. In addition, the present survey, aimed at describing the communication and visiting habits during the first pandemic wave, was conducted while Italy was facing the second wave. This, of course, involves a potential confounding factor, which needs to be added to the inherent risk of desirability and recall bias [57] due to the self-reporting nature of the study. Finally, while the reported number of patients treated by the participating ICUs is likely accurate, the number of visitors is certainly an estimate, as the presence/absence of family members is rarely reported in medical records.

## Conclusions

Family members of ICU patients had practically no access to the ICU during the first COVID-19 pandemic wave and daily remote communications served as a surrogate for family meetings. From an ethical and clinical point of view, restricting visits in the hospital/ICU can be justified only and exceptionally in case of PPE scarcity due to major risks for both the patients and the visitors. Currently, given the improved understanding of the prevention of COVID-19 transmission, the availability of PPE and the growing immunization due to mass vaccination, hospitals and ICUs should be responsibly reopened to visitors.

## Supplementary Information

**Additional file 1.** This additional file contains three additional tables, 1 additional figure and the translated version of the survey.

**Additional file 2.**

## Data Availability

The dataset used and/or analyzed during the current study are available from the corresponding author on reasonable request.

## Abbreviations

ARDS: Acute Respiratory Distress Syndrome
COVID-ICU: Intensive Care Unit dedicated to patients with COVID-19
COVID-19: Coronavirus disease − 19
ICU: Intensive Care Unit
mixed-ICU: Intensive Care Unit dedicated to both patients with and without COVID-19
Non-COVID-ICU: Intensive Care Unit dedicated to patients without COVID-19
OSM: Online supplementary material
PPE: Personal protective equipment
