# Correction: Communication and visiting policies in Italian intensive care units during the frst COVID-19 pandemic wave and lockdown: a nationwide survey

**DOI:** 10.1186/s12871-022-01813-3

**Published:** 2022-09-01

**Authors:** Thomas Langer, Francesca Carmela Depalo, Clarissa Forlini, Silvia Landini, Andrea Mezzetti, Paola Previtali, Gianpaola Monti, Carolina de Toma, Davide Biscardi, Alberto Giannini, Roberto Fumagalli, Giovanni Mistraletti, on behalf of the COMVISCOV group, Barbara Lissoni, Andrea De Martini, Nadia Mareto, Concetta Rossitto, Ugo Zummo, Martina Taverna, Patrizia Machieraldo, Mauro Navarra, Massimiliano Parlanti Garbero, Chiara Scaletti, Silvia Perno, Luca Amendolia, Giorgia Montrucchio, Deliana Veliaj, Giuseppe Barbarello, Maria Alesci, Luca Bolgiaghi, Davide Vailati, Angelo Pezzi, Enrico Boselli, Francesca Piccoli, Massimiliano Greco, Marco Gemma, Marco Resta, Stefania Crotti, Nicola Bottino, Chiara Abruzzese, Monica Savioli, Giuseppina Migliorino, Stefano Muttini, Michele Umbrello, Beatrice Borghi, Stefano Greco, Micaela Dizeo, Maurizio Bottiroli, Michele Giovanni Mondino, Manlio Prosepri, Giampaolo Casella, Francesco Curto, Matteo Zaniboni, Riccardo Giudici, Carlo Gentile, Michela Bombino, Roberto Rona, Barbara Cortinovis, Annalisa Benini, Leonello Avalli, Mario Tavola, Matteo Ferrario, Roberta Preda, Enzo Primerano, Gianluca Russo, Virginia Porta, Federico Valdambrini, Paola Fassini, Serena Orando, Eduardo Beck, Matteo Pedeferri, Giacomina Cogliati, Denise Testini, Benedetta Moroni, Vito Codeluppi, Patrizia Ruggeri, Elisa Milanesi, Mirko Belliato, Alessandra Besozzi, Mario Riccio, Silvia Zerbi, Davide Corbella, Francesco Ferri, Lorenzo Grazioli, Ezio Bonanomi, Matteo Giacomini, Noemi Sacchi, Cristian Codognola, Alessandra Ambrosini, Luca Guatteri, Matteo Subert, Gian Paolo Castelli, Massimo Borelli, Erica Venier, Loredana Dittura, Stefania Buttera, Roberto Bigai, Sandra Magnoni, Simon Rauch, Angelo Colombo, Giorgio Fullin, Caterina Donolato, Silvia Cattin, Veronica State, Enrico Redeghieri, Alessandro Russo, Simonetta Pastorini, Sandra Allena, Marina Munari, Federica Turchet, Mario Peta, Vincenzo De Santis, Cristina Scala, Francesca Facondini, Elisabetta Marangoni, Tania Tassinati, Chiara Zanzani, Emanuele Russo, Annamaria Marchio, Maria Barbagallo, Massimo Girardis, Paolo Taffache, Marco Mordacci, Matteo Vincenzi, Michele Pennica, Giovanna Bracciotti, Paola Iori, Davide Gambi, Iacopo Cappellini, Lara Vegnuti, Alessandra De Luca, Stefano Romagnoli, Giamila Mosti, Rossella Carla, Valeria Roticiani, Lorella Pelagalli, Ennio Fuselli, Emilio D’Avino, Massimo De Bellis, Giulia Gianni, Francesca Leonardis, Marzia Rossi, Rossana Lorusso, Eugenia Magnanimi, Sabrina Martelli, Floriana Baisi, Davide Balsamo, Virginia Cotticelli, Alessia Mattei, Ivano Farinelli, Teresa Riccini, Luisanna Cola, Antonella Jorio, Emanuele Iacobone, Roberta Domizi, Simone Pizzi, Armando Nasso, Romano Graziani, Anna Monaco, Manuela Manno, Carla Maria Ottelio, Michela Del Rio, Antonio Serra, Barbara Enna, Francesco Marco Loddo, Rita Galbiati, Serena Mellea, Michelle Brozzi Kimberly, Matteo Vissani, Francesco Massimo Romito, Laura Baccari, Nadia Zarrillo, Clelia Esposito, Patrizia Murino, Salvatore Notaro, Carmine Ausiello, Annachiara Marra, Carmela Policastro, Chiara Cafora, Giuseppe De Benedectis, Vincenzo Di Falco, Maria Sciddurlo, Giancarlo Negro, Paolo Vetuschi, Andrea Recchia, Rita Pasquariello, Rosalba Squillace, Antonio Ciambrone, Carmela Bencivenga, Melania Camiolo, Cristina Agozzino, Francesco Oliveri, Tiziana Notarrigo, Giacomo Castiglione, Antonella Mo, Laura Condorelli, Martina Favarato

**Affiliations:** 1 Department of Medicine and Surgery, University of Milan-Bicocca, Monza, Italy; 2 Department of Anesthesia and Intensive Care Medicine, Niguarda Ca’ Granda, Milan, Italy; 3 Azienda USL Toscana Centro, 118 Empoli, Empoli, Italy; 4 Department of Anesthesia and Intensive Care, ASST Santi Paolo e Carlo, San Paolo University Hospital, Milan, Italy; 5 Unit of Pediatric Anesthesia and Intensive Care, Children’s Hospital, ASST Spedali Civili, Brescia, Italy; 6 Department of Pathophysiology and Transplantation, University of Milan, Milan, Italy

**Correction: BMC Anesthesiol 22, 187 (2022)**

**https://doi.org/10.1186/s12871-022-01726-1**

Following publication of the original article [1], the authors identified a name error to one of the members of the COMVISCOV group. The name of Giorgia Montrucchio was mistakenly written.

The incorrect author name is: Giuseppe Montrucchio

The correct author name is: Giorgia Montrucchio

The author group has been updated above and the original article [1] has been corrected.
