# Supplementary material for: Communication and visiting policies in Italian intensive care units during the first COVID-19 pandemic wave and lockdown: a nationwide survey
Source: BMC Anesthesiol. 2022 Jun 17;22:187. doi: 10.1186/s12871-022-01726-1 (PMC9203262; doi:10.1186/s12871-022-01726-1)
Supplement: Supplementary file 1 — Additional file 1. This additional file contains three additional tables, 1 additional figure and the translated version of the survey. [file 12871_2022_1726_MOESM1_ESM.zip › 20220306 Supplementary information/Additional file 10ú║Table S2. Primers used for cloning..docx]

**Table S2 Primers used for cloning**

| Primer name | Sequence (5'-3') | Product length(bp) | TM(℃) |
| --- | --- | --- | --- |
| LOC109952131 | F1: CCTGCTGGTAACCTGTCTTTG | 450 | 53.6 |
|  | R1: TTAACCAGGTAAAGGTATTTTAATG |  |  |
| LOC109953466 | F1: CATTCCCTGATGTCAACGCAAC | 320 | 49 |
|  | R1: TTTATACCTTTTGTTACTTTGAAG |  |  |
| LOC109954337 | F1: TCATACCAGGTTTAGTGTGGAAG | 239 | 60 |
|  | R1: TCATACCAGGTTTAGTGTGGAAGC |  |  |
| LOC109954360 | F1: TTGCAGTTCCTCCCACTGTCAC | 424 | 60 |
|  | R1: GGACCACAGGTGGGGTCTCTG |  |  |
| LOC109958454 | F1: TCTCATTTGTTTTCTAGCCAGC | 454 | 55.8 |
|  | R1: GACTAATAACTGGGGTTTTATTAG |  |  |

F: forward primer; R: reverse primer.
