# Supplementary material for: Communication and visiting policies in Italian intensive care units during the first COVID-19 pandemic wave and lockdown: a nationwide survey
Source: BMC Anesthesiol. 2022 Jun 17;22:187. doi: 10.1186/s12871-022-01726-1 (PMC9203262; doi:10.1186/s12871-022-01726-1)
Supplement: Supplementary file 1 — Additional file 1. This additional file contains three additional tables, 1 additional figure and the translated version of the survey. [file 12871_2022_1726_MOESM1_ESM.zip › 20220306 Supplementary information/Additional file 11ú║Table S3. Primers used for qRT¿CPCR and incubation..docx]

Table S3 Primers used for qRT-PCR and incubation

| Primer name | Sequence (5'-3') | Product length(bp) | TM(℃) |
| --- | --- | --- | --- |
| LOC109952131 | F2: CTCTTGCTGGGACTGGTTGC | 154 | 57 |
|  | R2: GAGGAGGTGTAGGAGTTGGC |  |  |
| LOC109953466 | F2: ATGTCTGTCCTGTGGCTACT | 118 | 57 |
|  | R2: CTGCCTGAAAACACTTTATC |  |  |
| LOC109954337 | F2: GTCTTCACTGCAAACACCTG | 112 | 61 |
|  | R2: GCTTCTCCTTGGATTCATCT |  |  |
| LOC109954360 | F2: CAAGTCCCCTATTCATCCCC | 78 | 61 |
|  | R2: AACCACACGAAGTCTGCTCC |  |  |
| LOC109958454 | F2: ACCCCCAGCCACAGAATAAC | 126 | 57 |
|  | R2: TCCTCCTCCAGCCTCAGAAC |  |  |
| EF1αF1 | F1: CGCTGCTGTTTCCTTCGTCC | 102 | 59 |
|  | R1: TTGCGTTCAATCTTCCATCCC |  |  |
| RPL17F1 | F1: GTTGTAGCGACGGAAAGGGAC | 160 | 59 |
|  | R1: GACTAAATCATGCAAGTCGAGGG |  |  |
| U6F1 | F1: GGGTTACTTTGGTAGCACAT | 100 | 59 |
|  | R1: AAATTCGTGAAGCGTTCCTC |  |  |
| 18SF1 | F1: ACGAACAAGACTCCAGCAT | 129 | 59 |
|  | R1: CTGTGATGCCCTTAGATGT |  |  |
| znf207 | F1: GCAGGAAAGGAGAAGGAC | 192 | 59 |
|  | R1: CCAGCCACAGGAGGAATA |  |  |

F: forward primer; R: reverse primer.
