# Supplementary material for: Communication and visiting policies in Italian intensive care units during the first COVID-19 pandemic wave and lockdown: a nationwide survey
Source: BMC Anesthesiol. 2022 Jun 17;22:187. doi: 10.1186/s12871-022-01726-1 (PMC9203262; doi:10.1186/s12871-022-01726-1)
Supplement: Supplementary file 1 — Additional file 1. This additional file contains three additional tables, 1 additional figure and the translated version of the survey. [file 12871_2022_1726_MOESM1_ESM.zip › 20220306 Supplementary information/Additional file 1ú║Table S1. Quality inspection of transcriptome data..docx]

**Table S1. Quality inspection of transcriptome data**

| Sample name | Raw reads | Clean reads | clean bases | Error rate（%） | Q20（%） | Q30（%） | GC content（%） |
| --- | --- | --- | --- | --- | --- | --- | --- |
| IE1 | 122208478 | 117499704 | 17.62G | 0.01 | 97.93 | 94.62 | 47.75 |
| IE2 | 118497904 | 113779752 | 17.07G | 0.01 | 97.97 | 94.7 | 47.78 |
| IE3 | 112587104 | 108126242 | 16.22G | 0.01 | 97.98 | 94.71 | 47.86 |
| IL1 | 126654934 | 121287964 | 18.19G | 0.01 | 97.86 | 94.49 | 48.3 |
| IL2 | 130816696 | 124128046 | 18.62G | 0.01 | 97.82 | 94.43 | 47.94 |
| IL3 | 110418234 | 105558106 | 15.83G | 0.01 | 97.77 | 94.3 | 48.7 |
| IM1 | 133142504 | 127272988 | 19.09G | 0.01 | 98.06 | 94.91 | 48.1 |
| IM2 | 117994066 | 112682204 | 16.9G | 0.01 | 97.85 | 94.42 | 48.21 |
| IM3 | 128995190 | 124438390 | 18.67G | 0.01 | 97.76 | 94.26 | 47.95 |
| OV1 | 125771310 | 120693216 | 18.1G | 0.01 | 97.74 | 94.35 | 47.69 |
| OV2 | 116716772 | 112163932 | 16.82G | 0.01 | 97.87 | 94.46 | 48.12 |
| OV3 | 107260808 | 101886556 | 15.28G | 0.01 | 97.51 | 93.68 | 48.71 |
| TE1 | 119342536 | 115074368 | 17.26G | 0.01 | 97.91 | 94.61 | 47.06 |
| TE2 | 110474786 | 106618306 | 15.99G | 0.01 | 97.91 | 94.66 | 46.63 |
| TE3 | 114047316 | 109011776 | 16.35G | 0.01 | 97.54 | 93.69 | 48.06 |

(1) Raw reads: Count the raw sequence data, with four rows in a unit, and count the number of sequencing sequences in each file.

(2) Clean reads: The calculation method is the same as Raw Reads, except that the statistical data is the filtered sequencing data. Subsequent biological information analysis is based on Clean reads.

(3) Clean bases: Multiply the number of sequencing sequences by the length of the sequencing sequence and convert it to G as the unit.

(4) Error rate: average base sequencing error rate.

(5) Q20, Q30: Calculate the percentages of bases with Phred values greater than 20 and 30 to the total bases respectively.

(6) GC content: the percentage of the total number of bases G and C to the total number of bases.
