# Supplementary material for: Communication and visiting policies in Italian intensive care units during the first COVID-19 pandemic wave and lockdown: a nationwide survey
Source: BMC Anesthesiol. 2022 Jun 17;22:187. doi: 10.1186/s12871-022-01726-1 (PMC9203262; doi:10.1186/s12871-022-01726-1)
Supplement: Supplementary file 1 — Additional file 1. This additional file contains three additional tables, 1 additional figure and the translated version of the survey. [file 12871_2022_1726_MOESM1_ESM.zip › 20220306 Supplementary information/Additional file 7ú║Figure S3. KEGG enrichment analysis of DE-lncRNA trans-target genes..docx]

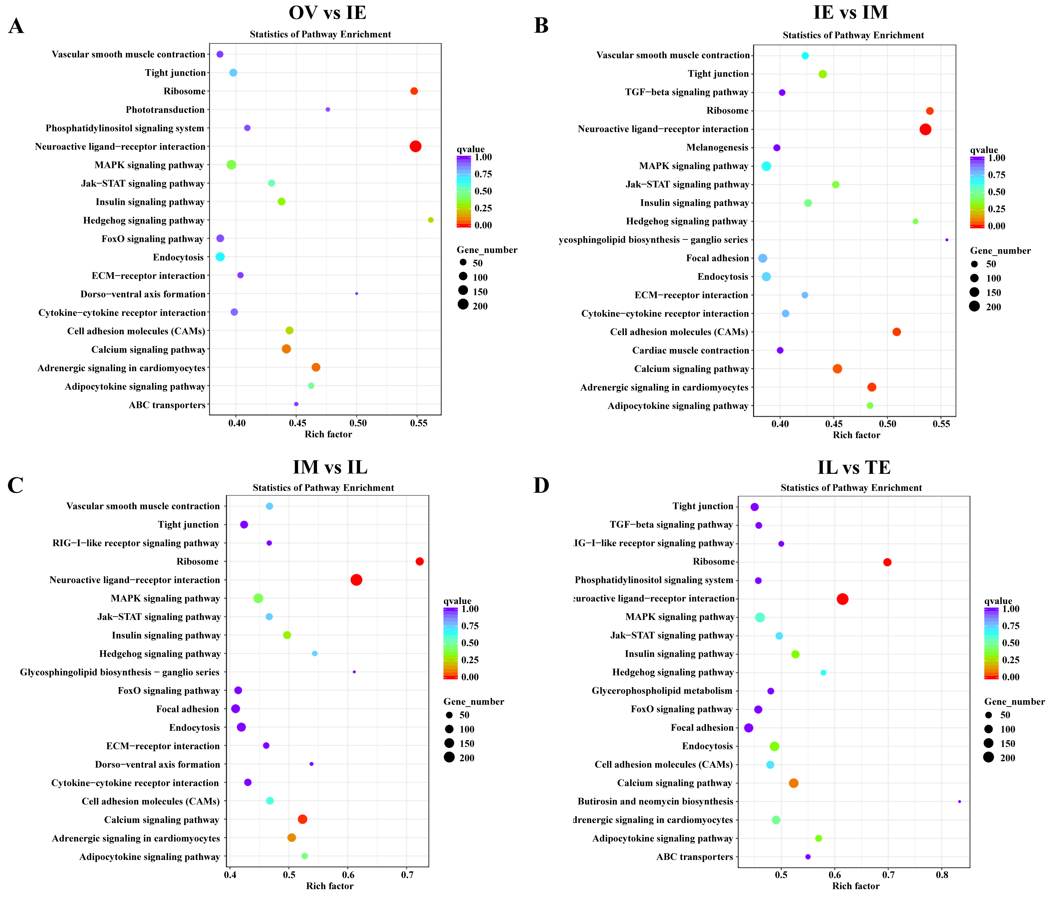


**Fig. S3 KEGG enrichment analysis of DE-lncRNA trans-target genes.** OV: ovary stage; IE: early intersexual stage; IM: mid-intersexual stage; IL: late intersexual stage; TE: testis stage.
