# Supplementary material for: Communication and visiting policies in Italian intensive care units during the first COVID-19 pandemic wave and lockdown: a nationwide survey
Source: BMC Anesthesiol. 2022 Jun 17;22:187. doi: 10.1186/s12871-022-01726-1 (PMC9203262; doi:10.1186/s12871-022-01726-1)
Supplement: Supplementary file 2 — Additional file 2. [file 12871_2022_1726_MOESM2_ESM.docx]

**Communication and visiting policies in Italian intensive care units during the first COVID-19 pandemic wave and lockdown: a nationwide survey.**

Thomas Langer^1,2^, Francesca Depalo^1,2^, Clarissa Forlini^1,2^, Silvia Landini^1^, Andrea Mezzetti^3^, Paola Previtali^2^, Gianpaola Monti^2^, Carolina de Toma^4^, Davide Biscardi^4^, Alberto Giannini^5^, Roberto Fumagalli^1,2^, Giovanni Mistraletti^4,6^

*on behalf of the COMVISCOV group*

***Online Supplementary Material***

^1^ Department of Medicine and Surgery, University of Milan-Bicocca, Monza, Italy

^2^ Department of Anesthesia and Intensive Care Medicine, Niguarda Ca' Granda, Milan, Italy

^3^118 Empoli, Azienda USL Toscana Centro, Empoli, Italy.

^4^ Department of Anesthesia and Intensive Care, ASST Santi Paolo e Carlo, San Paolo University Hospital, Milan, Italy.

^5^ Unit of Pediatric Anesthesia and Intensive Care, Children's Hospital, ASST Spedali Civili, Brescia, Italy

^6^ Department of Pathophysiology and Transplantation, University of Milan, Italy

**ADDITIONAL FIGURES**

**Figure E1. Geographical distribution of participating Intensive Care Units**

**
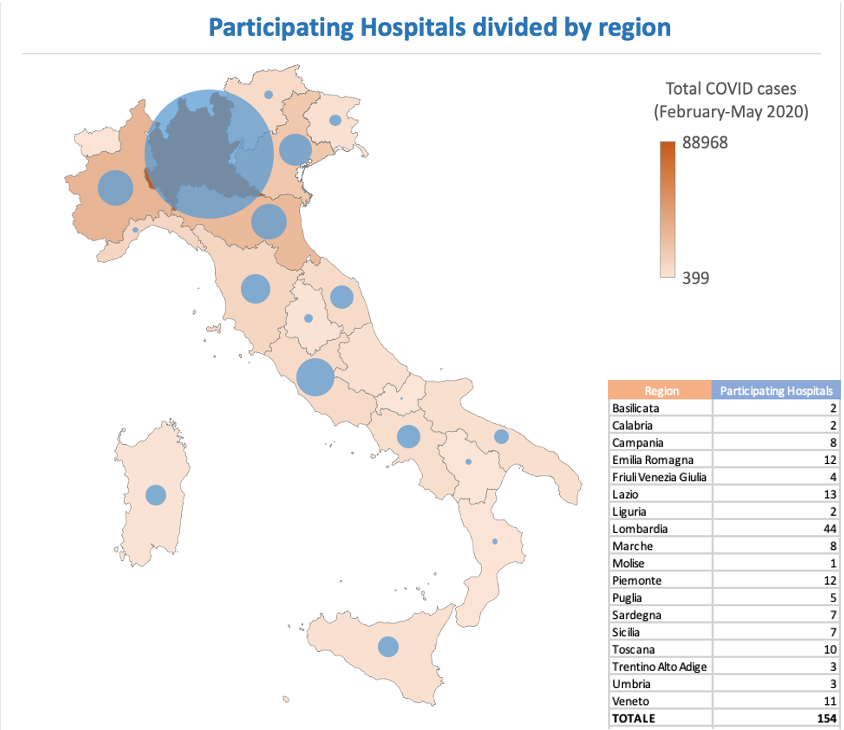
**

**Figure E1** represents Italy divided in its 20 regions and the geographical distribution of the participating ICUs. The size of the blue dot is proportional to the number of participants; the intensity of the red color of each region is proportional to the number of laboratory confirmed infections with SARS-CoV-2 during the first pandemic wave.

**Table E1. Differences between Responding and Non-Responding hospitals.**

|  | **Overall contacted hospitals**  n=290 | **Participating hospitals**  n=154  (53%) | N**onparticipating hospitals**  n=136 (47%) | **p-valu**e |
| --- | --- | --- | --- | --- |
| **University affiliated hospital -**  **no. (%)** | 63 (22) | 36 (23) | 27 (20) | 0.468 |
| **Trauma center -**  **no. (%)** | 78 (27) | 48 (31) | 30 (22) | 0.08 |
| **Hospital beds - no. (%)** |  |  |  | 0.04 |
| <250 | 100 (35) | 43 (28) | 57 (42) |  |
| 250-424 | 85 (29) | 49 (32) | 36 (27) |  |
| ≥ 425 | 105 (36) | 62 (40) | 43 (32) |  |
| **Dedicated room for family meetings - no. (%)**  - no. (%) | 236 (81) | 125 (81) | 111 (82) | 0.922 |
| **Visiting-hour policies** |  |  |  | 0.160 |
| ≤ 2 hours/day - no. (%) | 117 (40) | 56 (36) | 61 (45) |  |
| 3-6 hours/day - no. (%) | 83 (29) | 42 (27) | 41 (30) |  |
| 7-12 hours/day - no. (%) | 67 (23) | 40 (26) | 27 (20) |  |
| > 12 hours/day - no. (%) | 23 (8) | 16 (11) | 7 (5) |  |

**Table E2. Clinical psychology in adult ICUs**

|  | **Prepandemic** | **First pandemic wave** | **p-value** |
| --- | --- | --- | --- |
| Presence of clinical psychology service - no. (%) | 50 (31) | 74 (37) | 0.232 |
| Assistance to physicians in communication with families - no. (%) | 33 (20) | 61 (30) | 0.031 |
| Presence of clinical psychology service to assist patients/families - no. (%) | 47 (29) | 66 (33) | 0.430 |
| **Assistance to family members of awake**  **patients occurs through** | /47 | /66 | <0.001 |
| Direct interviews - no. (%) | 40 (85) | 22 (33) |  |
| Phone calls - no. (%) | 11 (23) | 52 (79) |  |
| Assisting family members during patient visit - no. (%) | 19 (40) | 14 (21) |  |
| **Assistance to awake patients occurs through** |  |  | 0.001 |
| Direct interviews | 35 (74) | 29 (44) |  |
| Indirect interviews through physician | 8 (17) | 32 (50) |  |
| Patient assistance during family visits | 13 (28) | 11 (17) |  |
| **Assistance to patients after discharge** | 31 (66) | 48 (73) | 0.439 |
| **Assistance to family members after discharge** | 28 (60) | 51 (77) | 0.043 |

**Table E3 Changes in communication habits in pediatric ICUs**

|  | **Prepandemic** | **First pandemic wave** | **p-value** |
| --- | --- | --- | --- |
| **Communication with families occurs** |  |  |  |
| Daily - no. (%) | 7 (100) | 7 (100) |  |
| **Use of electronic devices for physician - family communication - n (%)** | 6 (86) | 6 (86) |  |
| **Kind of electronic device used** | /6 | /6 | 0.580 |
| Voice call - no. (%) | 5 (83) | 5 (83) |  |
| Video call - no. (%) | 1 (17) | 3 (50) |  |
| **Communication between patient and family occurs** |  |  |  |
| Daily - no. (%) | 7 (100) | 7 (100) |  |
| **Patient has free access to personal electronic devices** | 7 (100) | 7 (100) |  |
| **Presence of  clinical psychology service** | 6 (86) | 6 (86) |  |
| **Assistance to family members of awake**  **patients occurs through** |  |  | 0.857 |
| Direct interviews | 6 (100) | 5 (83) |  |
| Phone calls | 2 (33) | 4 (67) |  |
| Assisting family members during patient visit | 2 (33) | 3 (50) |  |
| **Permission to visit patients** |  |  |  |
| Daily | 7 (100) | 7 (100) |  |
| **Which family member come to visit** |  |  |  |
| mainly the same family member/members | 7 (100) | 7 (100) |  |
| **Physical contact** | 7 (100) | 5 (71) |  |
| **Patients who received visits/total patients %** |  | 100 |  |

# SURVEY TRANSLATION

## SECTION 1: Hospital organization

In this section, we will ask you some information about the ICU where you currently work or have worked. The PRE-COVID-19 phase is the period prior February 2020. The COVID-19 phase is the period from February to May 2020.

1. Specify the hospital where you are working: _________________________________
2. Indicate the ICU you belong to (answer NONE if you didn’t work in the ICU during the PRE-COVID-19 phase): ___________________________________________________
3. Was the ICU where you worked in during the COVID-19 phase already active before the COVID-19 emergency?
   - Yes
   - No
4. Was the ICU where you worked in during the COVID-19 phase the same ICU where you had worked during the PRE-COVID-19 phase?
   - Yes
   - No
   - I didn’t work in an ICU
5. The ICU where you worked in during the COVID-19 phase was dedicated to patients:
   - positive for SARS-CoV-2
   - negative for SARS-CoV-2
   - both
6. How many ICUs are active in your hospital? (for the COVID-19 phase please include both COVID-19 and no-COVID-19 ICUs)
   - PRE-COVID-19 phase: ___(*number*)____
   - COVID-19 phase: ___(*number*)_____
7. How many ICU beds are there in your hospital? (PRE-COVID-19 phase): ___(*number*)____
8. Did your hospital need to increase the number of available ICU beds to take on the COVID-19 emergency?
   - Yes
   - No
9. What was the maximum number of occupied ICU beds (COVID 19 or not) in your hospital during the COVID-19 phase? ___(*number*)_____
10. What was the maximum number of ICU beds occupied by SARS-CoV-2 patients in your hospital? ___(*number*)_____
11. How many beds were available in your ICU during the PRE-COVID-19 phase? (Answer ZERO if you didn’t work in an ICU during the PRE-COVID-19 phase)
12. How many beds were available in your ICU during the COVID-19 phase?___(*number*)_____
13. How many patients were treated in your ICU during the COVID-19 phase?

- <5
- 5-10
- 11-20
- 21-30
- 31-40
- 41-50
- 51-60
- 61-70
- 71-80
- 81-90
- 91-100
- >100 (specify the number)

1. How many medical specialists were present in your ICU for the morning shift? (Hired residents should be considered as specialist doctors).

- PRE-COVID-19: ___(*number)*_____
- COVID-19: ___(*number*)_____

1. How many medical specialists were present in your ICU for the afternoon shift? (Hired residents should be considered as specialist doctors).

- PRE-COVID-19: ___(*number)*_____
- COVID-19: ___(*number*)_____

1. How many medical specialists were present in your ICU for the night shift? (Hired residents should be considered as specialist doctors).

- PRE-COVID-19: ___(*number)*_____
- COVID-19: ___(*number*)_____

1. Did your ICU also include Anesthesiology and Intensive Care residents during the PRE-COVID-19 phase? (Hired residents should be considered as specialist doctors)
   - Yes
   - No
   - I didn’t work in an ICU
2. Did your ICU also include Anesthesiology and Intensive Care residents during the COVID-19 phase? (Hired residents should be considered as specialist doctors).

- Yes
- No
- I didn’t work in an ICU

1. Did your hospital increase the ICU medical staff during the COVID-19 phase?

- Yes
- No

1. If the answer to the previous question was “Yes”, the increase was carried out by:
   - Hiring new specialist in Anesthesiology and Intensive Care
   - Hiring Anesthesiology and Intensive Care residents
   - Moving anesthetists from the OR
   - Moving specialist of different medical disciplines
2. Did your hospital increase the ICU nursing staff during the COVID-19 phase?

- Yes
- No

1. If the answer to the previous question was “Yes”, the increase was carried out by:
   - Hiring new nurses
   - Moving nurses from the OR
   - Moving nurses from other surgical and medical departments

## Section 2: Communication between patients and family members

In this section, we will ask information about how communications between patients and family members were managed.

1. During the PRE-COVID-19 phase, patients with a state of consciousness compatible with listening (*e.g.* extubated, tracheostomized during weaning) are allowed to communicate directly with their families, also through the use of electronic devices (*e.g.* mobile phone)?
   - Yes
   - No
   - I didn’t work in an ICU
2. During the COVID-19 phase, patients with a state of consciousness compatible with listening (*e.g.* extubated, tracheostomized during weaning) are allowed to communicate directly with their families, also through the use of electronic devices (*e.g.* mobile phone)?
   - Yes
   - No
   - I didn’t work in an ICU
3. How often patients could communicate with their family members during the PRE-COVID-19 phase?
   - Always (at least once a day)
   - Often (2-3 times per week)
   - Seldom (once a week)
   - Never
   - I didn’t work in an ICU
4. How often patients could communicate with their family members during the COVID-19 phase?
   - Always (at least once a day)
   - Often (2-3 times per week)
   - Seldom (once a week)
   - Never
5. In your ICU, do you have any electronic devices to facilitate communication between patients and family members? (PRE-COVID-19 phase)
   - Yes
   - No
   - I didn’t work in an ICU
6. In your ICU, do you have any electronic devices to facilitate communication between patients and family members? (COVID-19 phase)
   - Yes
   - No
7. What electronic devices did you use to facilitate direct communication between patients and their families? (PRE-COVID-19 phase):
   - Cellphone (audio calls only)
   - Smartphone (video calls)
   - Tablet (video calls)
   - Other
   - None
8. What electronic devices did you use to facilitate direct communication between patients and their families? (COVID-19 phase):
   - Cellphone (audio calls only)
   - Smartphone (video calls)
   - Tablet (video calls)
   - Other
   - None
9. Patients with a state of consciousness compatible with listening are allowed to use electronic devices (e.g. extubated, tracheostomized during weaning) to communicate directly with their family members ? (PRE-COVID-19 phase)
   - Yes
   - No
10. Patients with a state of consciousness compatible with listening are allowed to use electronic devices (e.g. extubated, tracheostomized during weaning) to communicate directly with their family members ? (COVID-19 phase)
    - Yes
    - No
11. How did you manage the use of electronic devices by patients to communicate with their family members? (PRE-COVID-19 phase)
    - Unrestricted (patients or family members could call whenever they want)
    - Restricted (medical staff managed electronic devices)
12. How did you manage the use of electronic devices by patients to communicate with their family members? (COVID-19 phase)?
    - Unrestricted (patients or family members could call whenever they want)
    - Restricted (medical staff managed electronic devices)

## Section 3: Communication between medical personnel and family members

In this section we will ask information about how communications between medical personnel and family members were managed.

1. Communication between medical staff and family members took place: (PRE-COVID-19 phase)
   - Always (at least once a day)
   - Often (2-3 times per week)
   - Seldom (once a week)
   - Never
   - I didn’t work in an ICU
2. Communication between medical staff and family members took place: (COVID-19 phase)
   - Always (at least once a day)
   - Often (2-3 times per week)
   - Seldom (once a week)
   - Never
3. Your ICU, had electronic devices available to facilitate the communication between medical staff and family members (PRE-COVID-19 phase)?
   - Yes
   - No
   - I didn’t work in an ICU
4. Your ICU, had electronic devices available to facilitate the communication between medical staff and family members ? (COVID-19 phase)
   - Yes
   - No
5. Specify which electronic devices you used to facilitate the communication between medical staff and family members (PRE-COVID-19 phase):
   - Mobilephone (audio calls only)
   - Smartphone (video calls)
   - Tablet (video calls)
   - Other
   - None
6. Specify which electronic devices you used to facilitate the communication between medical staff and family members (COVID-19 phase):
   - Mobilephone (audio calls only)
   - Smartphone (video calls)
   - Tablet (video calls)
   - Other
   - None
7. Who usually gave clinical news to family members during the PRE-COVID-19 phase?
   - usually the same person
   - usually a restricted set of people
   - the doctor in charge of the patient
8. Who usually gave clinical news to family members during the COVID-19 phase?
   - usually the same person
   - usually a restricted set of people
   - the doctor in charge of the patient
9. The person who gave clinical news to family members was (PRE-COVID-19 phase):
   - An experienced doctor (somebody that habitually communicates with patients’ families)
   - A non-experienced doctor (somebody that seldom or never communicates with patients’ families)
   - Both of the above
10. The person who gave clinical news to family members was (PRE-COVID-19 phase):
    - An experienced doctor (somebody that habitually communicates with patients’ families)
    - A non-experienced doctor (somebody that seldom or never communicates with patients’ families)
    - Both of the above
11. Who received usually clinical news? (PRE-COVID-19 phase):
    - mostly the same family member (*e.g.* wife, husband, son, daughter)
    - mostly a restricted set of family members (*e.g.* wife/husband and son/daughter)
    - Whoever showed up among family members
    - Whoever showed up among family members and friends
12. Who usually received clinical news? (COVID-19 phase):
    - mostly same family member (e.g. wife, husband, son, daughter)
    - mostly a restricted set of family members (e.g. wife/husband and son/daughter)
    - Whoever showed up among family members
    - Whoever showed up among family members and friends
13. As a doctor responsible for communicating news about a patient’s health, I believe that understanding the emotional state of a patient and their family members does not have any impact on the outcome of their treatment.

Do you agree with the above statement?

- I agree
- I neither agree nor disagree
- I disagree
- I cannot answer

1. Managing the relationship with a patient’s family members is an integral part of its treatment.

Do you agree with the above statement?

- I agree
- I neither agree nor disagree
- I disagree
- I cannot answer

1. As a doctor responsible for communicating news about a patient’s health, I do not allow myself to be emotionally involved in relationships with my patients and their families because it compromises the effectiveness of the treatment.

Do you agree with the above statement?

- I agree
- I neither agree nor disagree
- I disagree
- I cannot answer

## Section 4: The role of clinical psychology

In this section, we will ask information about the collaboration between medical personnel, family members and psychological support services.

1. Did your ICU have a psychological support specialist or clinical psychology service to assist medical staff during communications with patients’ family members (PRE-COVID-19 phase)?
   - Yes
   - No
   - I didn’t work in an ICU
2. Did your ICU have a psychological support specialist or clinical psychology service to assist medical staff during communications with patients’ family members (COVID-19 phase)?
   - Yes
   - No
3. Did your ICU have a psychological support specialist or clinical psychology service to assist patients and their family members (PRE-COVID-19 phase)?
   - Yes
   - No
   - I didn’t work in an ICU
4. Did your ICU have a psychological support specialist or clinical psychology service to assist patients and their family members (COVID-19 phase)?
   - Yes
   - No
   - I didn’t work in an ICU
5. How does the psychological support specialist interact with patients’ family members (PRE-COVID-19 phase)?
   - Direct communication: the psychological support specialist talks directly with family members
   - Indirect communication: the psychological support specialist helps the ICU medical personnel in talking with family members, but doesn’t interact with them directly
   - Both: the psychological support specialist talks directly with family members and also supports the medical personnel
   - None of the above
6. How does the psychological support specialist interact with patients’ family members (COVID-19 phase)?
   - Direct communication: the psychological support specialist talks directly with family members
   - Indirect communication: the psychological support specialist helps the ICU medical personnel in talking with family members, but doesn’t interact with them directly
   - Both: the psychological support specialist talks directly with family members and also supports the medical personnel
   - None of the above
7. Which of the following methods were used by the psychological support specialist to assist family members of patients capable of hearing (PRE-COVID-19 phase)?
   - Face-to-face talk with family members
   - Phone call with family members
   - Assisting family members while visiting the patient
8. Which of the following methods were used by the psychological support specialist to assist family members of patients capable of hearing (COVID-19 phase)?
   - Face-to-face talk with family members
   - Phone call with family members
   - Assisting family members while visiting the patient
9. Which of the following methods were used by the psychological support specialist to assist conscious patients (e.g. extubated patients, tracheostomy patients in protracted waning)? (PRE-COVID-19 phase)
   - Face to face talk with the patient
   - Indirect talk with the patient carried out through the responsible doctor
   - Assisting the patient during visits with family members
10. Which of the following methods were used by the psychological support specialist to assist conscious patients (e.g. extubated patients, trachesotomy patients in protracted waning) ? (COVID-19 phase)
    - Face to face talk with the patient
    - Indirect talk with the patient carried out through the responsible doctor
    - Assisting the patient during visits with family members
11. Did the psychological support specialist keep assisting patients after they were discharged from the ICU to a standard ward? (PRE-COVID-19 phase)
    - Yes
    - No
12. Did the psychological support specialist keep assisting patients after they were discharged from the ICU to a standard ward? (COVID-19 phase)
    - Yes
    - No
13. Did the psychological support specialist keep assisting family members after a patient was discharged from the ICU to a standard ward? (PRE-COVID-19 phase)
    - Yes
    - No
14. Did the psychological support specialist keep assisting family members after a patient was discharged from the ICU to a standard ward? (COVID-19 phase)
    - Yes
    - No

## Section 5: Visits

In this section, we will ask information about how visits from family members to hospitalized patients were managed.

1. Visits to patients by family members were carried out during hospitalization? (PRE-COVID-19 phase)
   - Yes
   - No
   - I didn’t work in an ICU
2. Visits to patients by family members were carried out during hospitalization? (COVID-19 phase)
   - Yes
   - No
   - I didn’t work in an ICU
3. How many hours a day were patients' relatives allowed to access in your ICU? (PRE-COVID 19)
   - Visits were not allowed
   - 1-2 hours per day
   - 3-6 hours per day
   - 7-12 hours per day
   - 13-24 hours per day
   - I didn’t work in an ICU
4. How often visits were allowed in your ICU? (PRE-COVID-19 phase)
   - Always (once a day)
   - Often (2-3 times per week, scheduled beforehand)
   - Seldom (only for exceptional circumstances, e.g. patients about to die)
   - Never
5. How often visits were allowed in your ICU? (COVID-19 phase)
   - Always (once a day)
   - Often (2-3 times per week, scheduled beforehand)
   - Seldom (only for exceptional circumstances, e.g. patients about to die)
   - Never
6. Who were the people allowed to visit a patient in your ICU? (PRE-COVID-19 phase)
   - mostly the same family member (e.g. wife, husband, son, daughter)
   - mostly a restricted set of family members (e.g. wife/husband and son/daughter)
   - Whoever showed up among family members
   - Whoever showed up among family members and friends
   - Visits were not allowed in my ICU
7. Who were the people allowed to visit a patient in your ICU? (COVID-19 phase)
   - mostly the same family member (e.g. wife, husband, son, daughter)
   - mostly a restricted set of family members (e.g. wife/husband and son/daughter)
   - Whoever showed up among family members
   - Whoever showed up among family members and friends
   - Visits were not allowed in my ICU
8. During the COVID-19 phase, how many patients received at least one visit from a family member during their ICU length of stay?
   - 0
   - 1
   - 2
   - 3-5
   - 6-10
   - 11-15
   - 16-20
   - 21-30
   - More than 30 (specify the number)
9. Was physical contact between patients and family members allowed during the PRE-COVID-19 phase?
   - Yes
   - No
   - Visits were not allowed in my ICU
10. Was physical contact between patients and family members allowed during the COVID-19 phase?
    - Yes
    - No
    - Visits were not allowed in my ICU
11. Was the psychological support specialist present during visits from family members? (PRE-COVID-19 phase)
    - Always (present during every visit)
    - Seldom (only during visits for exceptional circumstances)
    - Never
    - Visits were not allowed in my ICU
12. Was the psychological support specialist present during visits from family members? (COVID-19 phase)
    - Always (present during every visit)
    - Seldom (only during visits for exceptional circumstances)
    - Never
    - Visits were not allowed in my ICU
13. Specify the reason/s why visits were not allowed during the COVID-19 phase (please answer even if some visits were allowed for a few patients)
    - ICU-specific rule
    - Hospital-wide interdiction
    - Refusal from family members
    - Self-quarantine by family members
    - Mandatory quarantine enforced by the national Government
14. How satisfied do you think the patients' families are satisfied with your way of managing the doctor-patient-family relationship?
    - Very satisfied
    - Somewhat satisfied
    - Very little satisfied
    - Not at all satisfied
    - I am not sure
